# Single‐Atom Catalysts on C_3_N_4_: Minimizing Single Atom Pt Loading for Maximized Photocatalytic Hydrogen Production Efficiency

**DOI:** 10.1002/anie.202416453

**Published:** 2025-01-02

**Authors:** Nawres Lazaar, Siming Wu, Shanshan Qin, Abdessalem Hamrouni, Bidyut Bikash Sarma, Dimitry E. Doronkin, Nikita Denisov, Hinda Lachheb, Patrik Schmuki

**Affiliations:** ^1^ Department of Materials Science WW4-LKO Friedrich-Alexander-University of Erlangen-Nuremberg Martensstrasse 7 91058 Erlangen Germany; ^2^ Research Laboratory of Catalysis and Materials for the Environment and Processes LRCMEP (LR19ES08) University of Gabès, Faculty of Sciences of Gabès (FSG) University Campus Erriadh City 6072 Gabès Tunisia; ^3^ Laboratoire des Substances Naturelles Institut National de Recherche et d'Analyse Physico-chimique, INRAP Pôle Technologique de Sidi Thabet 2020 Tunisia; ^4^ Laboratoire de Chimie de Coordination (LCC),CNRS Université de Toulouse, INPT, UPR 8241 205 route de Narbonne 31077 Toulouse Cedex 4 France; ^5^ Institute of Catalysis Research and Technology KIT Hermann-von Helmholtz Platz 1 76344 Eggenstein-Leopoldshafen Germany; ^6^ Regional Centre of Advanced Technologies and Materials Šlechtitelů 27 78371 Olomouc Czech Republic

**Keywords:** dark deposition, Pt single atoms, C_3_N_4_, H_2_ evolution, photocatalysis

## Abstract

The use of metal single atoms (SAs) as co‐catalysts on semiconductors has emerged as a promising technology to enhance their photocatalytic hydrogen production performance. In this study, we describe the deposition of very low amounts of Pt SAs (<0.1 at %) on exfoliated graphitic carbon nitride (C_3_N_4_) by a direct Pt−deposition approach from highly dilute chloroplatinic acid precursors. We find that − using this technique−a remarkably low loading of highly dispersed Pt SAs (0.03 wt %) on C_3_N_4_ is sufficient to achieve a drastic decrease in the overall charge transfer resistance and a maximized photocatalytic efficiency. The resulting low‐loaded Pt SAs/C_3_N_4_ provides a H_2_ production rate of 1.66 m mol/h/mg Pt, with a remarkable stability against agglomeration; even during prolonged photocatalytic reactions no sign of light‐induced Pt agglomerations can be observed. We ascribe the high performance and stability to the site‐selective, stable coordination of Pt within the C_3_N_4_ structure. Notably the H_2_ production rate of the low‐loaded Pt SAs surpasses the activity of Pt SAs deposited by other techniques or nanoparticles at comparable or even higher loading – the optimized Pt SAs decorated C_3_N_4_ show ≈5.9 times higher rate than Pt NP decorated C_3_N_4_.

## Graphical abstract

Pt single atoms are successfully anchored onto C_3_N_4_ through a reactive deposition approach, achieving optimal positioning of Pt atoms in the most stable and active configurations. This strategy results in maximized photocatalytic H_2_ production efficiency at a low Pt loading of approximately 0.03 wt %.

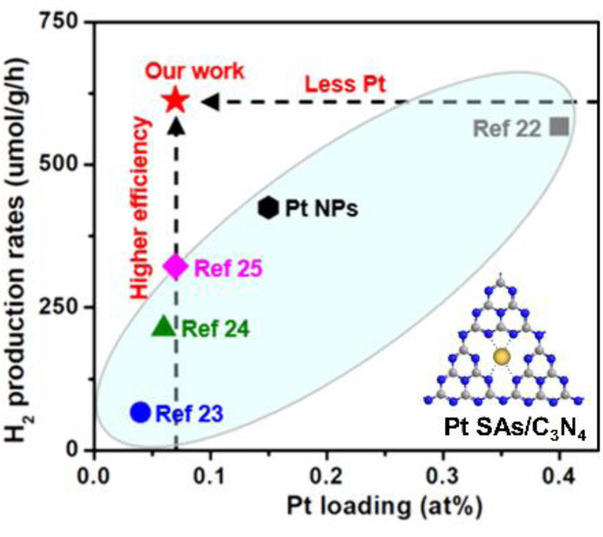

## 1 Introduction

Photocatalytic water splitting using semiconductor materials has gained significant attention as a promising approach for sustainable hydrogen production.[^1^, ^2^, ^3^, ^4^] Most of the pioneering work has been performed on titanium dioxide (TiO_2_) due to its excellent stability, abundance, and favorable band structure for the water splitting reactions.[^5^, ^6^] Meanwhile extensive exploration of a wide array of other semiconductors has taken place, and in recent years, particularly graphitic carbon nitride (C_3_N_4_) has attracted high attention for similar beneficial features as TiO_2_ but with the crucial advantage of a band‐gap in the visible light range. The latter allows for a much higher absorption of solar light and thus conceptually a much higher hydrogen production efficiency.[^7^, ^8^, ^9^, ^10^] Nonetheless, the actual hydrogen production efficiency of many semiconductors including TiO_2_ or C_3_N_4_ falls short of expectations, mainly due to a slow charge transfer and reaction kinetics at the semiconductor‐solution interface.[^11^, ^12^, ^13^]

This issue can be addressed by application of suitable charge‐transfer co‐catalysts. Particularly platinum (Pt), decorated or incorporated as nanoparticles, nanoclusters, or single atoms (SAs), has been extensively explored on many semiconductors. In the case of C_3_N_4_, the use of Pt nanoparticles anchored to the surface represents a most conventional approach to reach reasonable hydrogen evolution efficiencies.[^14^, ^15^] However, the reliance on substantial amounts of this expensive noble metal (typically 2–5 wt %) has been a major barrier to practical deployment due to high costs and the inefficient use of Pt atoms.[^16^, ^17^, ^18^, ^19^] This is a main driver for the use of nanoclusters and eventually SAs in photocatalysis[^20^, ^21^]–SAs can provide a maximized atom utilization efficiency at a maximized catalytic efficiency.

In the case of C_3_N_4_, there is already a large body of synthesis approaches for the production of Pt nanoparticles and Pt SA‐loaded materials. In situ approaches as well as post‐synthesis techniques have been reported, namely using photodeposition or atomic layer deposition, that are often followed by calcination steps.[^20^, ^22^, ^23^, ^24^, ^25^, ^26^] Such SA‐loaded C_3_N_4_ photocatalysts providean efficient enhancement of photocatalytic H_2_ production, and remarkable light‐to‐H_2_ conversion efficiencies have been achieved. Nevertheless, these approaches operate with a comparably high loading of Pt (often due to the fact that the deposition techniques lead to a mixed loading of SAs and nanoparticles). While a number of experiments and theoretical approaches point to a most effective metal loading at N4 coordination sites,[^23^, ^27^, ^28^, ^29^] most deposition techniques provide a (somewhat) random loading of Pt on C_3_N_4_ surfaces. It may be speculated that a significant amount of the Pt deposited is present in a non‐active or non‐ideal surface configuration on the C_3_N_4_ surface.^[30]^ Therefore, it would be highly desirable to find an approach that leads to Pt SAs deposited at most active sites which then would minimize the Pt loading while maximizing the efficiency. This would optimize the photocatalytic reaction, not only in terms of H_2_ production but would also yield a maximized Pt ultilization.

In this context, it is noteworthy that recently it was shown on defective TiO_2_ surfaces that a simple so‐called “reactive” deposition approach from very dilute Pt‐precursor solutions can lead to well anchored SAs with a remarkably high efficiency at a remarkably low Pt loading.[^31^, ^32^, ^33^, ^34^, ^35^] It has been postulated that the approach is self‐guiding^[36]^ i.e. dilute Pt‐acid precursors react at most reactive sites which then leads “self‐guided” to Pt SAs loaded (coordinated) in a most active surface configuration.^[36]^ In the present work, we explore the feasibility to apply the very same principle to C_3_N_4_, i.e. to harvest from aqueous solutions minute amounts of Pt in the form of single atoms by a direct reaction of C_3_N_4_ with chloroplatinic acid. After reaction, we explore the functionality of the deposited Pt SAs as a co‐catalyst in photocatalytic H_2_ generation and we evaluate the conditions for a maximum H_2_ production efficiency. We show that this approach of loading Pt SAs on exfoliated g‐C_3_N_4_ (Pt SAs/C_3_N_4_) can provide a C_3_N_4_ photocatalyst that with a minimum of Pt loading of 0.03 wt % can yield remarkably high H_2_ production efficiencies as well as Pt utilization efficiencies. If the activity is normalized to the Pt‐loading, unprecedented H_2_ production efficiencies (1.66 mmol/h/mg Pt) can be reached. Furthermore, the Pt SAs loaded by this approach show a very high stability of the Pt SAs – even after 24 h of reaction not any change in coordination or agglomeration of the Pt SAs could be observed. The high activity and stability of Pt SAs/C_3_N_4_ are attributed to the robust coordination of Pt SAs within the C_3_N_4_ structure, representing the most stable configuration and the most effective catalytic site. The findings are not only of a high scientific interest in terms of identifying ideal pairs of semiconductors and co‐catalysts, and optimizing anchoring of SAs on substrates, but also in terms of the most cost‐effective use of precious metals in photocatalysis.

## 2 Results and Discussion

**Figure 1:**
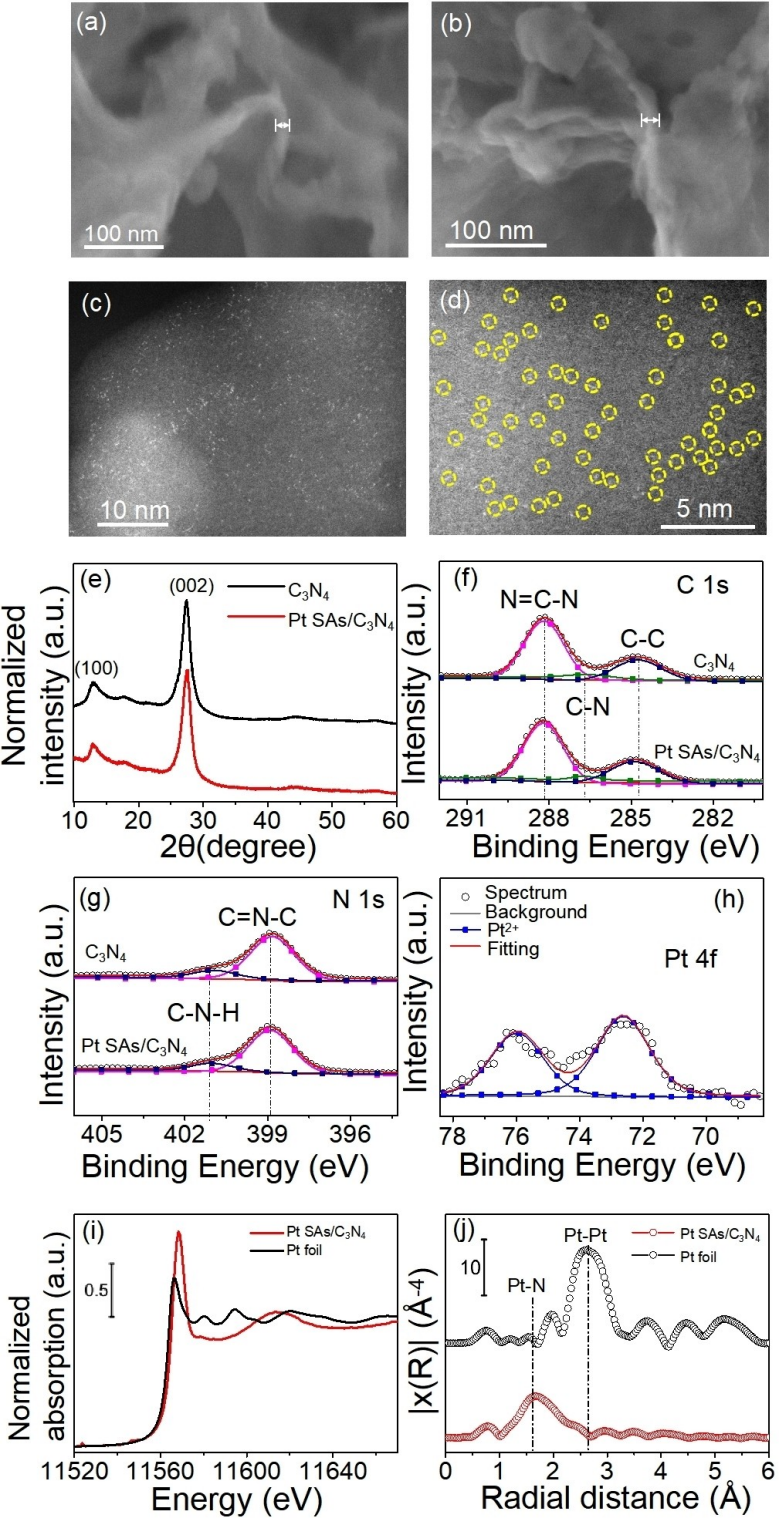
(a) SEM image of bare C_3_N_4_, (b) SEM image of Pt SAs/C_3_N_4_, (c),(d) HAADF STEM images of Pt SAs/C_3_N_4_, (e) XRD and XPS spectra of (f) C 1s, (g) N 1s of C_3_N_4_ and Pt SAs/C_3_N_4_, (h) Pt 4 f XPS spectrum, (i) Normalized XANES spectra at the Pt L_3_ edge and (j) Fourier transform EXAFS spectra (R‐space) of Pt SAs/C_3_N_4_.

We first prepare delaminated g‐C_3_N_4_ powder according to well‐established literature procedures by synthesis from an equimolar mixture of melamine and dicyandiamide followed by thermal delamination at 500 °C for 2 hours.[^37^, ^38^, ^39^] The decoration of Pt SAs on C_3_N_4_ is further carried out by a “spontaneous deposition” approach – that is, reaction of the C_3_N_4_ surface with an aqueous H_2_PtCl_6_ solutions in the concentration range of 10 mM‐0.05 mM (more details are given in the experimental section).^[31]^ Figure 1a shows an SEM image of the delaminated g‐C_3_N_4_ and Figure 1b shows an SEM image of the delaminated g‐C_3_N_4_ after Pt deposition– in this case loading was carried out in a 2 mM H_2_PtCl_6_ solution. The delaminated C_3_N_4_ and the Pt SA loaded C_3_N_4_ (Pt SAs/C_3_N_4_) are present as a sheet‐like structure with a layer thickness of 18 nm (Figure 1a,b). No obvious difference of the morphology is observed after Pt deposition compared with the bare C_3_N_4_ sample. High‐angle annular dark‐field scanning transmission electron microscopy (HAADF‐STEM) images of Pt SAs/C_3_N_4_ with different magnifications are presented in Figure 1c,d. No Pt agglomeration nor nanoparticles can be observed in Figure 1c. Figure 1d, taken at a higher magnification, further highlights Pt atoms are evidently present as well‐separated individual SAs, which are marked by yellow circles. EDX mapping confirms the uniform dispersion of Pt SAs on the C_3_N_4_ structure (Figure S1). The density of Pt SAs is approximately 9.6×10^5^ μm^−2^, as estimated from HAADF‐STEM images such as in Figure S2.

The XRD patterns of C_3_N_4_ and Pt SAs/C_3_N_4_ is shown in Figure 1e. Both samples (before and after Pt loading) show the characteristic peaks of g‐C_3_N_4_, consistent with previous research works.^[40]^ Specifically, two distinct peaks are visible at 2θ values around 12.9° and 27.7° that are attributed to the (100) repeating motif of tri‐s‐triazine and the (002) interlayer stacking of the conjugated aromatic system, respectively. Noteworthy, no diffraction peaks related to metallic Pt can be identified for the Pt SAs/C_3_N_4_ sample.

We further carried out X‐rayabsorption spectroscopy (XAS)^[41]^ in terms of X‐ray absorption near‐edge spectra (XANES) and X‐ray absorptionfine structure (EXAFS) and Diffuse reflectance infrared Fourier transform spectroscopy (DRIFTS)^[42]^ experiments to investigate the oxidation state of Pt and its relevant coordination enviroment. The normalized XANES spectra measured at the PtL_3_‐edge (11.564 keV) suggest that Pt SAs/C_3_N_4_ sample has positively charged Pt centers (Figure 1i).^[22]^ The extended EXAFS analysis and the corresponding Fourier transformed (FT) radial distribution function show a predominant featureat 1.6 Å (without phase correction) which originates from a light atom such as N in the Pt−N coordination (Figure 1j).[^22^, ^29^] The CO‐DRIFTS spectra of Pt SAs/C_3_N_4_ (Figure S3) show obvious CO vibrational frequency at 2120 cm^−1^, which is characteristic of linearly bonded CO over Pt single sites.[^43^, ^44^] These results confirm that Pt atoms are well‐isolated within the C_3_N_4_ structure, with minimal agglomeration or clustering, attributed to the Pt−N coordination.

X‐ray photoelectron spectroscopy (XPS) was employed to obtain compositional information of the C_3_N_4_ and Pt SAs/C_3_N_4_ samples (Figure 1f‐h, Figure S4). As shown in Figure 1f, the C 1s spectrum of both samples can be fitted into three peaks at 288.1 eV, 286.7 eV and 284.7 eV, which are assigned to N=C‐N, C−N and C−C, respectively.^[22]^ Furthermore, the fitted N 1s spectrum contains two peaks at 398.9 and 401.1 eV, corresponding to C=N−C, and C−N−H, respectively.^[22]^ No obvious difference is observed in the C 1s and N 1s spectrum after Pt SAs deposition. Figure 1h shows the XPS spectra of the Pt 4 f region for the Pt SAs/C_3_N_4_ sample. The peak positions for Pt 4f_7/2_ at ≈72.6 eV and Pt 4f_5/2_ at ≈76 eV correspond well to a Pt oxidation state of δ^+^ (δ≈2). I.e. the Pt^4+^ precursor, [Pt(Cl)_6_]^2−^, has been reduced to Pt^δ+^ during the process.^[45]^ The fact that no Cl is detected, as shown in the Cl 2p XPS in Figure S4, furthermore indicates that the Cl^−^ coordination is completely lost during the surface reaction of Pt and its coordination to C_3_N_4_. These reaction characterisitics are well in line with the introduction of chloroplatinic acid and attachment of Pt SAs on TiO_2_. I.e. a successful reductive coupling reaction of the Pt species with the C_3_N_4_ has taken place; i.e. Pt is not simply adsorbed on the surface, but the [Pt(Cl)_6_]^2−^ has reacted with suitable surface sites (reduction of Pt^4+^ to Pt^δ+^and loss of Cl‐coordination).^[46]^ Further evaluation of the relative concentrations from XPS shows 0.07 at% Pt for the deposition from the 2 mM H_2_PtCl_6_ precursor (Table S1). For comparison also, Pt nanoparticles were deposited following a classic photodeposition approach from literature.^[43]^ For this sample SEM images presented in Figure S5b show distinct Pt nanoparticles which are clearly visible on the C_3_N_4_ surface, and from the corresponding XPS Pt 4 f region (Figure S5a), we find Pt at a concentration of 0.14 at%. The position of the Pt 4 f peaks at 70.6 eV and 73.9 eV are in good agreement with literature data for metallic Pt^0^.^[43]^

**Figure 2:**
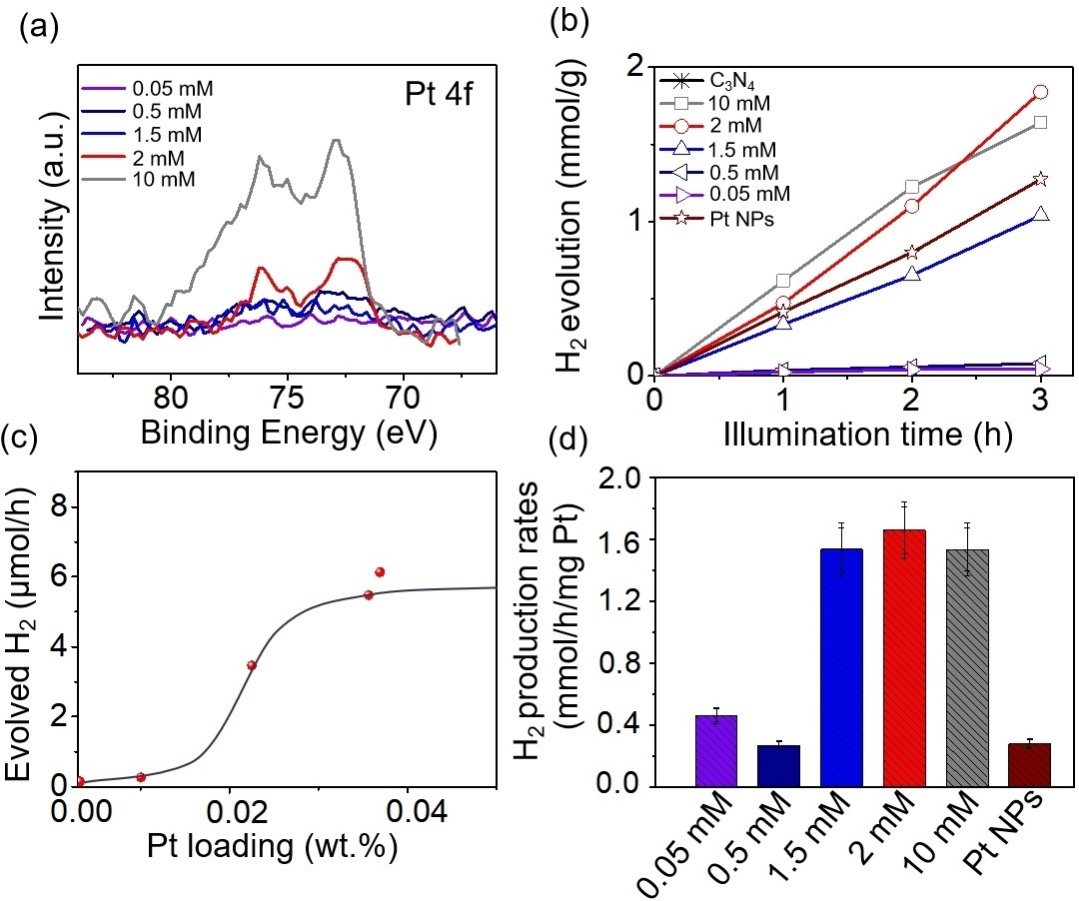
(a) Pt 4 f XPS spectra of Pt SAs/C_3_N_4_ prepared with different concentrations of Pt precursor, (b) Photocatalytic H_2_ evolution of Pt SAs/C_3_N_4_ prepared with different concentrations of Pt precursor and Pt NPs decorated C_3_N_4_, (c) Evolved H_2_ at different Pt SAs loading, and (d) Normalized H_2_ evolution rates for the different samples.

Figure 2a shows the Pt 4 f region for C_3_N_4_ loaded with different concentrations of Pt precursor (H_2_PtCl_6_), ranging from 10 mM to 0.05 mM, used for SA deposition. Under all deposition conditions, no peaks of metallic Pt are observed. Instead, Pt SAs (Pt^δ+^) are successfully decorated on all samples with Pt 4 f peaks at 72.6 eV and 76 eV. Only at the highest concentration of 10 mM is there some concentration of Pt^4+^, evident from the Pt 4 f peaks at 75 eV and 78.5 eV, which indicates presence of some adsorbed non‐reacted precursor. With increasing Pt precursor concentration an increase in Pt loading is observed as summarized in Table S1. Notably, even at the highest Pt precursor concentration of 10 mM, which results in a loading of 0.23 at%, no Pt nanoparticles are observed on C_3_N_4_, as evident from the SEM image in Figure S6.

To additionally determine the bulk loading of Pt we used by atomic absorption spectroscopy (AAS) and the results are shown in Figure S7 and Table S2. An increasing Pt loading is observed from 0.05 mM to 2 mM, which is consistent with the XPS data. At higher precursor concentration (10 mM), the deposited amount does not further increase, indicating that saturation of reactive Pt SA loading on C_3_N_4_ is reached at a Pt loading of 0.03 wt %. This finding is in line with previous work on TiO_2_, where also Pt SA loading reaches a plateau at a distinct loading concentration corresponding to a saturation of reactive anchoring points.^[33]^

We then studied the photocatalytic H_2_ generation of the C_3_N_4_ samples (using a LED 365 nm 65 mW/cm^2^, as a light source, and 10 vol % triethanolamine as a sacrificial agent; further experimental details are described in the Experimental section). Results for all samples are shown in Figure 2b. Within the concentration range of 0.05 mM to 10 mM, the loading increases from 0.003 wt % to 0.03 wt % and the photocatalytic H_2_ production activity is dependent on the Pt SA loading. Evidently a maximum H_2_ production efficiency is already reached for sample synthesized with 2 mM H_2_PtCl_6_, which corresponds to very low loading amount of 0.03 wt % (from AAS data in Table S2). For those samples, the loading of Pt SAs is sufficient to provide a fully effective co‐catalytic effect.In Figure 2c the photocatalytic activity is plotted *vs* the Pt SA loading to better assess the optimal amount of Pt SAs needed as cocatalyst on C_3_N_4_ for maximum co‐catalytic effect. Initially, an increase in H_2_ production is observed with increasing Pt loading (using Pt precursor from 0.05 mM to 2 mM), followed by a plateau where further increases in Pt loading do not significantly enhance the activity. Thus, a very low loading of ~0.03 wt % is sufficient to achieve the highest H_2_ production amount. Importantly, this low loading for maximized efficiency is specific to the reactive deposition method we developed. Other methods either achieve similar Pt SA loadings with significantly lower activity or require much higher loadings to achieve comparable activity, which will be discussed later. Evidently also the Pt SAs outperform Pt NPs by photodeposition, which have a much higher loading (0.15 wt %, Table S2). This observation clearly emphasizes the highly effective beneficial impact of the Pt SAs deposited by the present approach.

To further evaluate and compare the efficiency of Pt as a cocatalyst in the forms of SAs and NPs, we normalized the data in Figure 2b with the Pt loading (Table S2), and the results are shown in Figure 2d. Evidently, a maximum mass‐specific photocatalytic efficiency, yielding an H_2_ production rate of 1.66 mmol/h/mg Pt, is achieved with 2 mM Pt (0.03 wt %). This value is 5.9 times higher than that obtained by the classic photodeposition of Pt NPs on C_3_N_4_ (and significantly higher than any results obtained for common literature procedures, as we will show later). The turnover frequency (TOF) of Pt SAs decorated C_3_N_4_ (0.03 wt % Pt loading) reaches 324 h^−1^. I.e. the Pt species deposited on C_3_N_4_ by this simple dark deposition approach show a remarkable activity (and this without any further thermal treatments).

**Figure 3:**
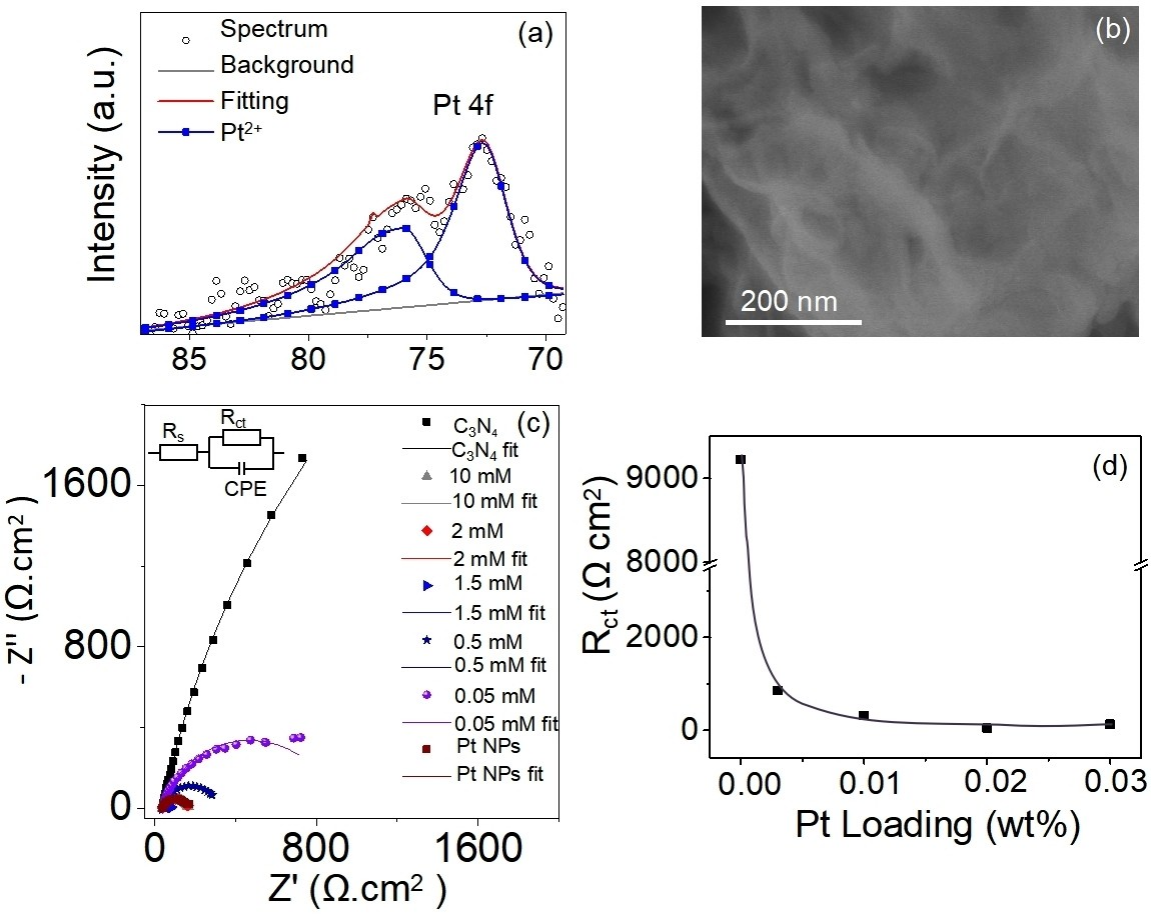
(a) Pt 4 f XPS spectrum and (b) SEM image of Pt SAs/C_3_N_4_ after illumination, (c) EIS plots of C_3_N_4_, Pt SAs/C_3_N_4_ and Pt NPs/C_3_N_4_ at the voltage −0.5 V (vs.Ag/AgCl) in 0.1 M Na_2_SO_4_ aqueous electrolyte.The equivalent circuit model used for fitting is depicted in the inset of Figure 3c and (d) *R*_ct_ vs. Pt loading plot of Pt SAs/C_3_N_4_ samples.

Particularly remarkable is the robustness of the Pt^δ+^ state attained by reactive deposition. For many photocatalytic systems, illumination of surface coordinated Pt SA (e.g. oxygen coordinated Pt^δ+^ on TiO_2_) leads to rapid photoinduced reduction to Pt^0^ and agglomeration as metallic Pt nanoparticles.^[22]^ In our case Pt remains as SAs with δ≈2 after illumination (Figure 3a). This aligns with observations from SEM images (Figure 3b) where after illumination on the active surface no Pt nanoparticles can be observed. Notably, the Pt^2+^ state persists even after prolonged irradiation of 24 hours, with a slight decrease in activity possibly due to the loss of loosely bound Pt species and the depletion of the sacrificial agent. (Figure S8). This excellent stability of Pt SAs can be explained by arobust coordination of the Pt atoms within the C_3_N_4_ structure. In literature,[^23^, ^48^] namely sites that provide an N_4_ coordination in triazines can lead to very stable Pt (II) species due to chelation effects, aromatic stabilization and favorable electronic properties. For example, Xiong et al. investigated the coordination between Pt and N using XANES and XPS and found that g‐C_3_N_4_ can form coordination bonds with Pt^2+^ through different N sites.^[29]^ Particularly the Pt^2+^‐N_4_ coordination state was identified as most stable and active, and as such advantageous for water splitting.^[49]^ Additionally, Zhang et al. proved the electron injection from Pt atom to N atom in Pt SAs decorated C_3_N_4_ upon light irradiation by in situ XPS.^[22]^ This may suggest that the high activity and stability of Pt SAs/C_3_N_4_ is due to dynamic evolution of Pt SAs within the C_3_N_4_ structure, and promote the efficieny of electron transfer to Pt SA. To elucidate the effect of Pt SA loading on the electrochemical nature of the C_3_N_4_ photocatalyst – namely the effects on the charge transfer characteristics, we additionally performed electrochemical impedance spectroscopy (EIS) measurements in 0.1 M Na_2_SO_4_. Figure 3c shows the Nyquist plots for electrode formed from bare C_3_N_4_, and C_3_N_4_ deocrated with different amount of Pt SAs as well as for C_3_N_4_ decorated with Pt NPs (zoomed‐in spectra in Figure S9). A classic Randles equivalent circuit was employed for fitting the Nyquist plots (Figure 3c, inset). Already a semiquantitative assessment shows the radius of the fitted curve for Pt SAs/C_3_N_4_ to be significantly smaller compared to bare C_3_N_4_, indicating strong drop in charge transfer resistance upon Pt SA loading. Quantitative fitting data are summarized in Table S4, confirming a 70 times drop in *R*_ct_. In Figure 3d, the *R*_ct_ values are plotted against the Pt SA loading. Remarkably, even a very low loading of Pt SAs (0.02 wt %) can drastically improve the charge transfer from C_3_N_4_ to the electrolyte. This is remarkably well in line with the low loading to reach maximum efficiency in photocatalytic H_2_ production. In contrast, for Pt nanoparticles (Pt NPs) a seven times higher loading of 0.15 wt % is needed to achieve a similar reduction in charge transfer resistance. These results indicate the high efficiency of minor amounts of Pt SAs in strongly mediating the charge transfer resistance of C_3_N_4_.

Incident photon‐to‐current conversion efficiency (IPCE) measurement was also carried out to evaluate the photoelectrochemical properties of Pt SAs/C_3_N_4_. As shown in Figure S10, obvious photocurrent is observed for C_3_N_4_ and Pt SAs/C_3_N_4_ in the wavelength range of 300–450 nm, and the determined band gap is ≈2.7 eV (Figure S10, inset), which is typical for C_3_N_4_. Pt SAs/C_3_N_4_ show similar IPCE value with bare C_3_N_4_, indicating that the light absorption characteristic is hardly affected by the deposition of Pt SAs due to the low loading of Pt.We find well in line with literature that the layers show an n‐type semiconductor behavior (Figure S11) with an increase in the photocurrent at potentials close to the flat‐band potential, reflecting the strong beneficial effect of Pt SAs on the charge transfer also under illumination conditions.

**Figure 4:**
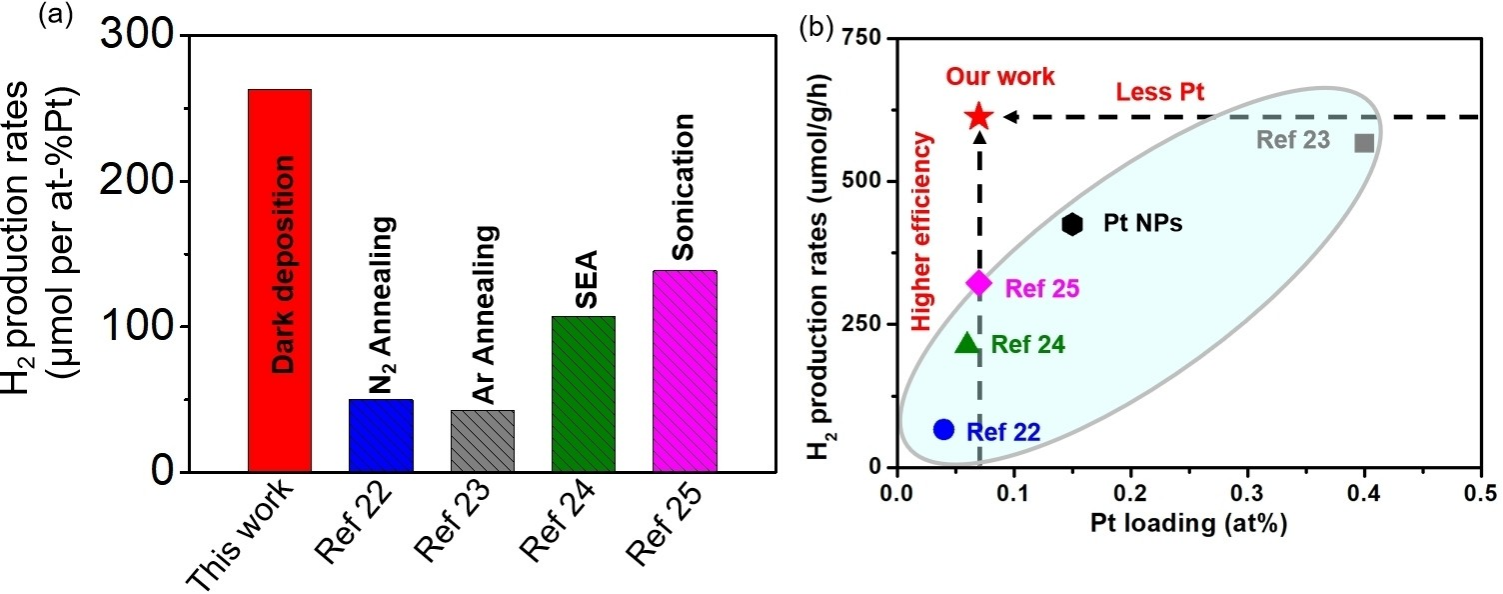
Comparison of (a) Normalized H_2_ evolution rates and (b) Comparsion of Pt loading and H_2_ production rates of Pt NPs decorated C_3_N_4_ and Pt SAs decorated C_3_N_4_ prepared with different procedures.

The extraordinary performance of the dark deposition loaded Pt SAs on the exfoliated C_3_N_4_ structure is particularly apparent, when this structure is compared to other Pt‐loaded C_3_N_4_ structures that were previously used and reported for photocatalytic H_2_ generation. In order to exclude the effect of different reactor geometries, light sources, etc, the comparison of Pt SAs decorated C_3_N_4_ samples were tested under the same conditionsas our our C_3_N_4_ samples. A comparison of the photocatalytic H_2_ evolution results of all samples are shown in Figure 4a. Not only do Pt SAs decorated on C_3_N_4_ by our reactive deposition approach achieve the highest photocatalytic H_2_ production activity but evenmore they achieve this with a remarkably low loading of Pt SAs (Table S3). An overall comparison of Pt loading and H_2_ production rates is summarized in Figure 4b. In some studies with similar amount of Pt SAs loaded,[^22^, ^24^, ^25^] the H_2_ production rates are much lower, indicating the higher efficiency of our Pt SAs or a higher efficiency of our Pt SAs with equally low amounts. Conversely, other studies achieve activity levels similar to ours, but require more than ten times the Pt SA loading.^[23]^ I.e. our reactive deposition approach, achieves unprecedented efficiency in the use of Pt SAs on C_3_N_4_, demonstrating that a very low loading is sufficient to maximize photocatalytic H_2_ production efficiency of such C_3_N_4_ based photocatalysts. This high efficiency of Pt SAs can be attributed to the nature of the deposition method: The reaction of the Pt‐precursor occurs at suitable reactive sites of C_3_N_4_ – these Pt decorated sites then are most efficient for charge transfer and catalysis. In this sense the process is self‐guiding: Pt is decorated where it is needed and where it is effective. Based on literature consideration some may consider Pt‐N_4_ configurations as this active site as they represent a most stable and effective catalytic coordination for Pt SAs within C_3_N_4_ structures.[^23^, ^27^, ^28^, ^29^] These sites become saturated at low Pt concentrations, indicating a comparably low site density. Nevertheless, this low amount of Pt SAs is sufficiently effective in reaching an optimal photocatalytic efficiency, thus eliminating the need for additional Pt.

## 3 Conclusions

In this study, we report a successful decoration of Pt SAs on exfoliated C_3_N_4_ through a reactive deposition approach, resulting in uniformly dispersed and highly stable Pt SAs. Remarkably, a very low loading of 0.03 wt % Pt SAs can lead to a maximized photocatalytic H_2_ production efficiency. A maximized H_2_ production rate of 1.66 mmol/h/mg Pt is achieved with Pt SAs/C_3_N_4_, far surpassing that of other SA deposition approaches on C_3_N_4_, as well as Pt nanoparticles decorated C_3_N_4_. Moreover, Pt SA deposited by our reactive deposition approach show superior stability in the photocatalytic H_2_ production process – no light‐induced SA agglomeration could be found even after prolonged illumination. Key effects of Pt SA loading is a strongly enhanced electron transfer even at very small loading by Pt SAs, together with their coordination within the C_3_N_4_ structure. Overall, the results show that reactive deposition, involving a self‐guided anchoring of Pt SAs at reactive C_3_N_4_ sites provides exceptionally active and stable co‐catalytic sites in C_3_N_4_ based photocatalysis.

## Conflict of Interests

The authors declare no conflict of interest.

4

## Supporting information

As a service to our authors and readers, this journal provides supporting information supplied by the authors. Such materials are peer reviewed and may be re‐organized for online delivery, but are not copy‐edited or typeset. Technical support issues arising from supporting information (other than missing files) should be addressed to the authors.

Supporting Information

## Data Availability

The data that support the findings of this study are available from the corresponding author upon reasonable request.
