# Supplementary material for: Single‐Atom Catalysts on C3N4: Minimizing Single Atom Pt Loading for Maximized Photocatalytic Hydrogen Production Efficiency
Source: Angew Chem Int Ed Engl. 2025 Jan 2;64(6):e202416453. doi: 10.1002/anie.202416453 (PMC11795736; doi:10.1002/anie.202416453)
Supplement: Supplementary file 1 — Supporting Information [file ANIE-64-e202416453-s001.pdf]

Angewandte Chemie
Eine Zeitschrift der Gesellschaft Deutscher Chemiker

Supporting Information

Single-Atom Catalysts on C_3N_4: Minimizing Single Atom Pt Loading for Maximized Photocatalytic Hydrogen Production Efficiency

*N. Lazaar, S. Wu, S. Qin, A. Hamrouni, B. Bikash Sarma, D. E. Doronkin, N. Denisov, H. Lachheb, P. Schmuki**

Supporting Information

Single-Atom Catalysts on C_3N_4: Minimizing Single Atom Pt Loading for Maximized Photocatalytic Hydrogen Production Efficiency

*Nawres Lazaar,[1,2#] Siming Wu,[1#] Shanshan Qin,[1] Abdessalem Hamrouni,[2,3] Bidyut Bikash Sarma,[4] Dimitry E. Doronkin,[5] Nikita Denisov,[1] Hinda Lachheb[2] and Patrik Schmuki[1,6]**

[1]Department of Materials Science WW4-LKO, Friedrich-Alexander-University of Erlangen-Nuremberg, Martensstrasse 7, 91058 Erlangen, Germany

[2]Research Laboratory of Catalysis and Materials for the Environment and Processes LRCMEP (LR19ES08), University of Gabès, Faculty of Sciences of Gabès (FSG), University Campus Erriadh City, Gabès, 6072, Tunisia

[3]Laboratoire des Substances Naturelles, Institut National de Recherche et d'Analyse Physico-chimique, INRAP, Pôle Technologique de Sidi Thabet, 2020, Tunisia,

[4]Laboratoire de Chimie de Coordination (LCC), CNRS, Université de Toulouse, INPT, UPR 8241, 205 route de Narbonne, 31077 Toulouse Cedex 4, France

[5]Institute of Catalysis Research and Technology, KIT, Hermann-von Helmholtz Platz 1, 76344 Eggenstein-Leopoldshafen, Germany

[6]Regional Centre of Advanced Technologies and Materials, Šlechtitelů 27, 78371 Olomouc, Czech Republic

Email: schmuki@ww.uni-erlangen.de
[#]Equal Contribution

Experimental section:

Materials: Dicyandiamide ($C_2H_4N_4$, Alfa Aesar, 99%), Melamine ($C_3H_6N_6$, Alfa Aesar, 99%), $H_2PtCl_6.6H_2O$ (Sigma Aldrich, 99.995%), Triethanolamine (Sigma-Aldrich, ≥99%), Sodium Sulfate (Na_2SO_4 , carl Roth, ≥99%), Ethanol (C_2H_6O, Carl Roth, ≥99.8%), Nafion (Sigma-Aldrich,5wt% in mixture of lower aliphatic alcohols and water, contains 45% wate), Methanol (CH_3OH, Carl Roth, ≥99.9%)

Synthesis of graphitic carbon nitride (g-C_3N_4)

g-C_3N_4, was synthesized by a thermal polycondensation method using an equimolar mixture of melamine and dicyandiamide. The process involved, mixing the melamine and dicyandiamide in an agate mortar, placing the mixture in an alumina crucible with a coverlet and then calcining at 530 °C for 2 h in a muffle furnace with a heating rate of 5 °C/min. Subsequently, the resulting powders were washed with distilled water, filtered, and dried overnight at 100 °C. The obtained sample was then collected and loaded in an open crucible, calcined in air at 500 °C for 2 h. The as prepared product is labeled as C_3N_4.

Synthesis of Pt SAs/C_3N_4

Pt SAs/C_3N_4 was synthesized through a "dark deposition" method.[1]A suspension containing 60 mg of C_3N_4 and 30 mL of ultrapure water was purged with argon (Ar) for 15 min to remove the residual oxygen in the solution. Following this, an appropriate concentration of H_2PtCl_6 (10 mM, 2 mM, 1.5 mM, 0.5 mM and 0.05 mM) was added to the solution. The system was then closed and maintained in darkness within sealed quartz cells for 1h under continuous stirring. After the dark deposition process, the resulting powder underwent a washing procedure involving centrifugation and three consecutive rinses with deionized (DI) water. Following the washes, the powder was collected and subjected to drying at 70 °C overnight.

Synthesis of Pt NPs/C_3N_4

The reference sample was decorated with Pt nanoparticles by photodeposition method. Initially, 60 mg of C_3N_4 was added to a 30 mL triethanolamine solution containing 3 wt% Pt. The solution was purged with N_2 and exposed to 365 nm LED irradiation (600 mW/cm^2) for 6 h under continuous stirring. Following irradiation, the sample was washed, collected and dried overnight at 70 °C.

Photocatalytic H_2 evolution

The photocatalytic process was carried out in a sealed quartz reactor containing 10 mL 10 vol% Triethanolamine solution. To remove oxygen from the solution, the system was purged with argon (Ar) for 15 min. Then closed and irradiated for 3h with an LED (λ = 365 nm, power density of 65 mW/cm^2) under continuous stirring. The hydrogen production was detected each 1h by gas chromatography (GCMS-QO2010SE, SHIMADZU) equipped with a thermal conductivity detector (TCD).

Characterization

High-angle annular dark field scanning transmission electron microscopy (HAADF-STEM) images were obtained using probe-corrected thermo Fisher Scientific spectra 200. The electrothermal Atomization AAS is employed to accurately quantify the Pt loading in graphitic carbon nitride (g-C_3N_4) samples by using a graphite furnace. The analysis was conducted with a contra 600 spectrometer (Analytik Jena AG), equipped with a high-resolution Echelle double monochromator and a continuum radiation source (Xe lamp). The morphology and elemental composition of the photocatalysts were analyzed using a field-emission scanning electron microscope (SEM, Hitachi S-4800) that was equipped with energy dispersive X-ray spectroscopy (EDAX Genesis). The structural study was carried out on an X'pert Philips MPD diffractometer with a Panalytical X'celerator detector using a Cu Kα radiation source (λ = 1.54056 Å). The oxidation states and the chemical composition of the samples were investigated using X-ray photoelectron spectroscopy (XPS) (PHI 5600).

X-ray Absorption Spectroscopy (XAS): X-ray absorption spectra at Pt L_3-edge (11564 eV) were collected at the P65 beamline of the Deutsches Elektronen-Synchrotron (DESY), Hamburg, Germany. The incident energy was scanned with a Si (111) double crystal monochromator (DCM). For measuring the reference samples, pellets diluted with cellulose were used, and the measurements were conducted in transmission. For ex-situ measurements, the catalyst powderwas placed in a plastic sample holder (5 mm path length) without dilution.The measurements were conducted in fluorescence mode using a Hitachi Vortex-ME4 silicon drift detector. The processing of the XAS data (data reduction, alignment, normalization, background subtraction, Fourier transformation) has been performed using the Athena code (version0.9.26). EXAFS data was k^3-weighted andFourier transformed in the range of 2-11.5 Å^{-1}.

Diffuse reflectance infra-red spectroscopy (DRIFTS): In-situ CO-DRIFTS experiment was conducted on a VERTEX 70 FTIR spectrometer from Bruker equipped with an Mercury-Cadmumum Telluride (MCT) detector. A Harrick Praying Mantis optics and the Harrick high temperature cell with a flat CaF_2 window were used. The sample was measured in powder form. The catalyst was pre-treated by purging Ar and heating to 353 K for 1 h and then the spectra were collected under 1% CO/Ar for 1h in reflectance mode between 1000-4000 cm^{-1}. A 4 cm^{-1} spectral resolution was used. The spectra are reported in logarithm log(1/R) or in Kubelka-Munk. For the background spectra, 150 scans were collected and averaged under Ar flow and for the samples 150 scans were collected per measurement.

Photoelectrochemical studies

Photoelectrochemical measurements, specifically IPCE (Incident Photon-to-current Efficiency) and electrochemical impedance spectroscopy (EIS), as well as transient photocurrent studies were carried out in a three-electrode cell setup, comprising the working electrode (sample), a Pt foil counter electrode, and an Ag/AgCl reference electrode. The IPCE measurements were carried out in a 0.1 M Na_2SO_4 solution in H_2O:Methanol= 90:10 vol.%, while the EIS measurements were performed in a 0.1 M Na_2SO_4 aqueous solution. The working electrode was made by dispersing 1 mg of C_3N_4 photocatalysts in 20 μL of Nafion, 50 μL of H_2O and 50 μL of ethanol. The mixture was sonicated for 20 min, and then 20 μL of the dispersed solution was applied to a carbon electrode.

IPCE spectra were recorded in the 300-800 nm wavelength range using a 150 W Xenon arc lamp from LOT-Oriel Instruments, coupled with a Cornerstone motorized 1/8 m monochromator. The EIS measurements were performed using an electrochemical workstation Zahner IM6 (Zahner Elektrik, Kronach, Germany). The impedance response was recorded in the dark at -0.5 V (vs. Ag/AgCl) with a 5 mV potential perturbation. The data were analyzed and fitted using the "EIS Spectrum Analyzer" software to extract the electrical parameters of the C_3N_4 materials. Additionally, the transient photocurrent measurements were performed using 0.1 M Na_2SO_4 aqueous solution as the electrolyte. The potential was swept in the cathodic direction from 0V to -1V (Vs. Ag/AgCl) at a scan rate of 0.05 V/s.The illumination was cycled on and off every 30 seconds. For the working electrode, 1 mg of photocatalyst material was mixed with 10 μL of Nafion, 100 μL of H_2O and 100 μL of ethanol. After 5 min of sonication, 10 μL of the dispersion was applied to a glassy carbon electrode.

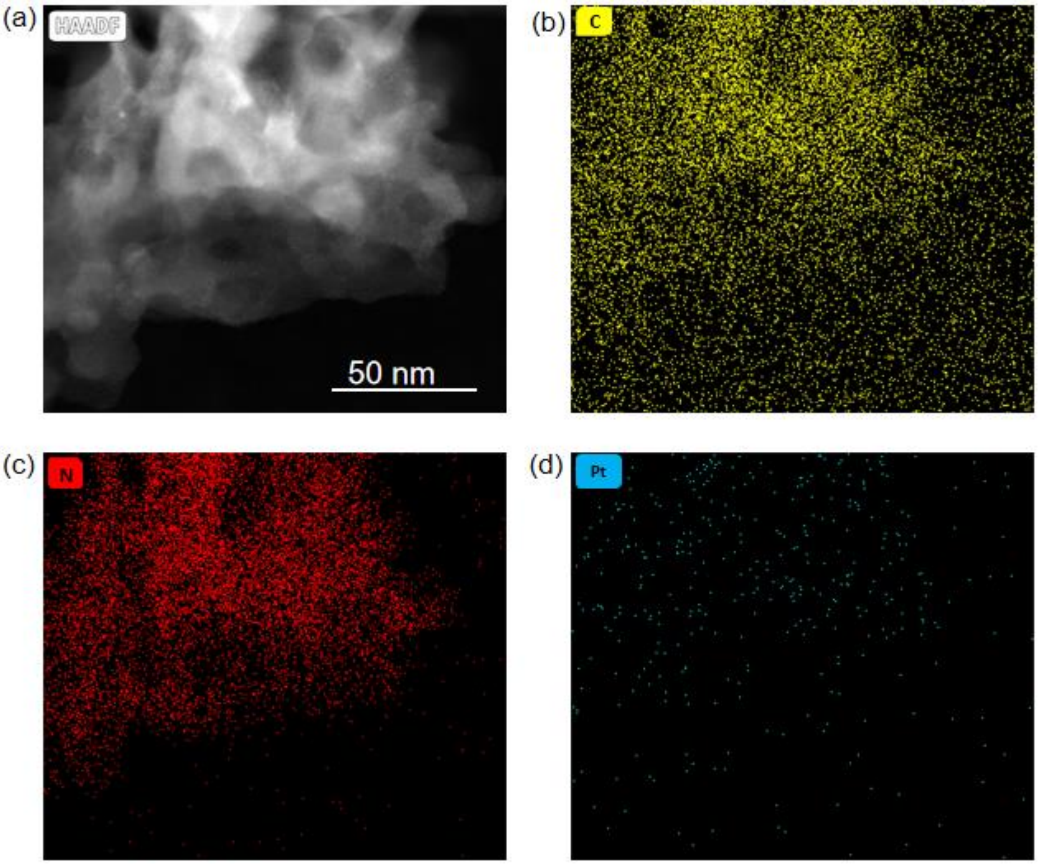

Figure S1.(a) HAADF-STEM image, (b)-(d) elemental mapping of (b) C, (c) N and (d) Pt of Pt SAs/C_3N_4.

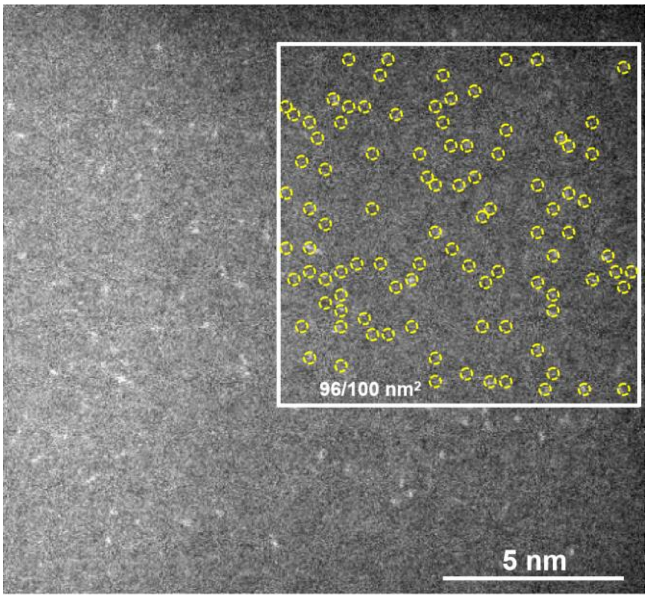

Figure S2. STEM image of Pt SAs/C_3N_4 (SA density = 9.6×10^5 μm^{-2}).

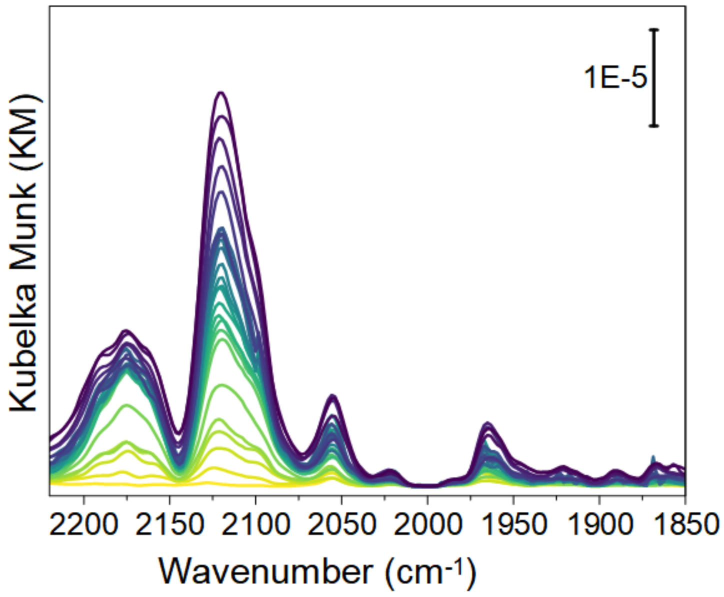

Figure S3. CO-DRIFTS spectra of Pt SAs/C_3N_4.

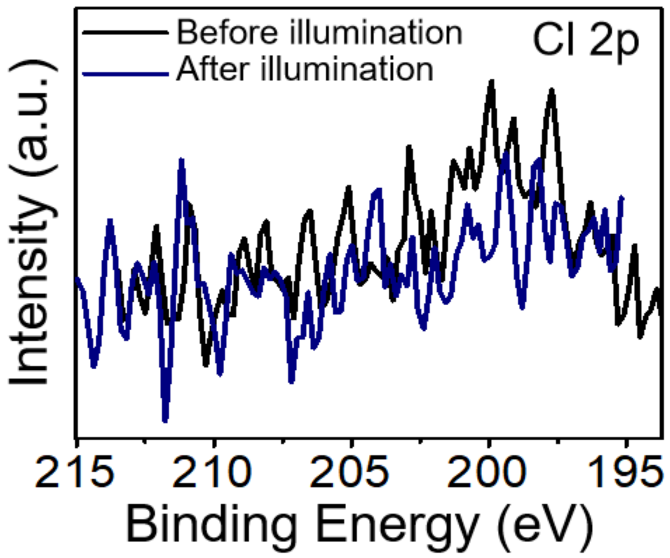

Figure S4. Cl 2p XPS spectra of Pt SAs/C_3N_4 before and after illumination.

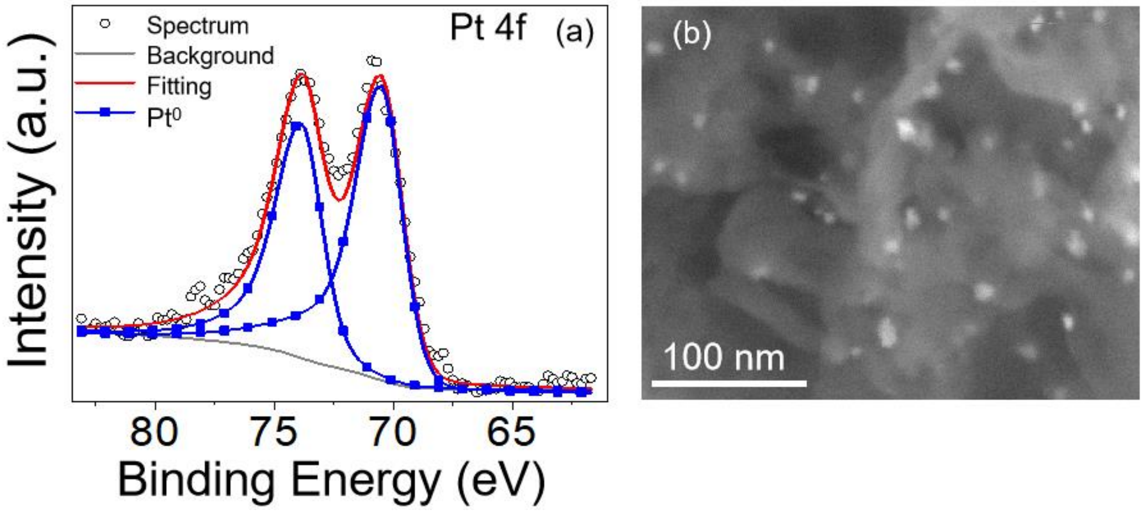

Figure S5. (a) Pt 4f XPS spectra and (b) SEM image of Pt NPs/C_3N_4 sample.

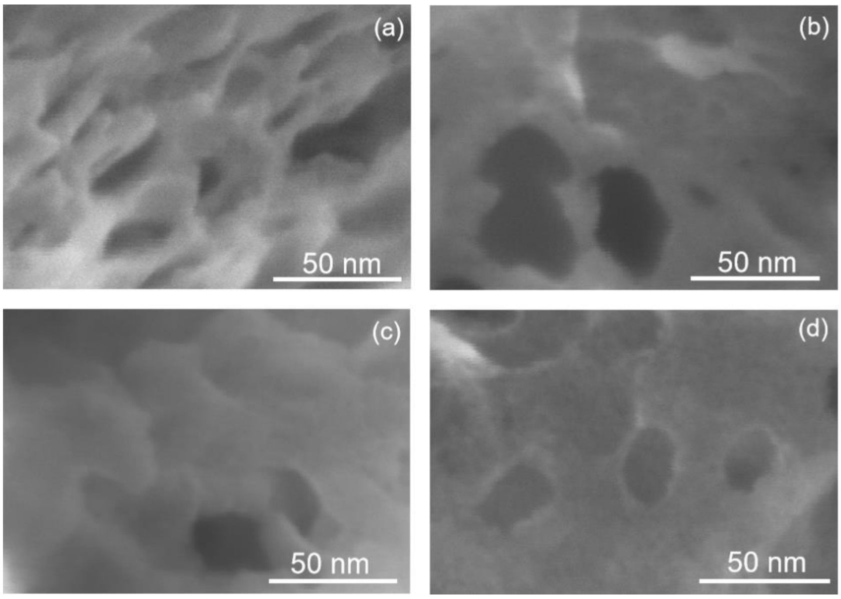

Figure S6. SEM images of Pt SAs/C_3N_4 prepared with (a) 10mM, (b) 1.5 mM, (c) 0.5mM and (d) 0.05 mM H_2PtCl_6 precursor.

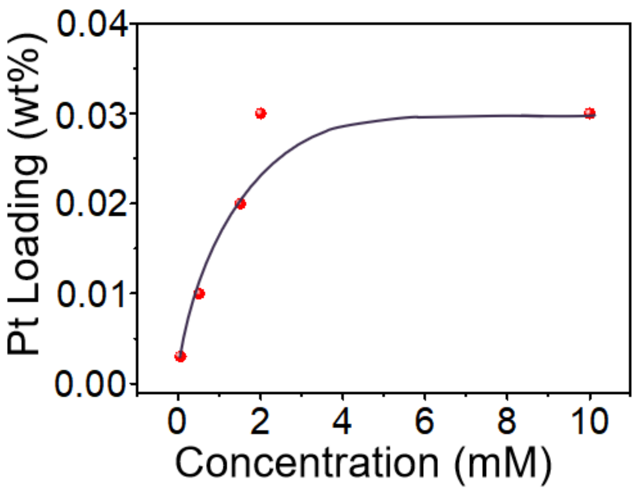

Figure S7. Pt loading vs. precursor concentration.

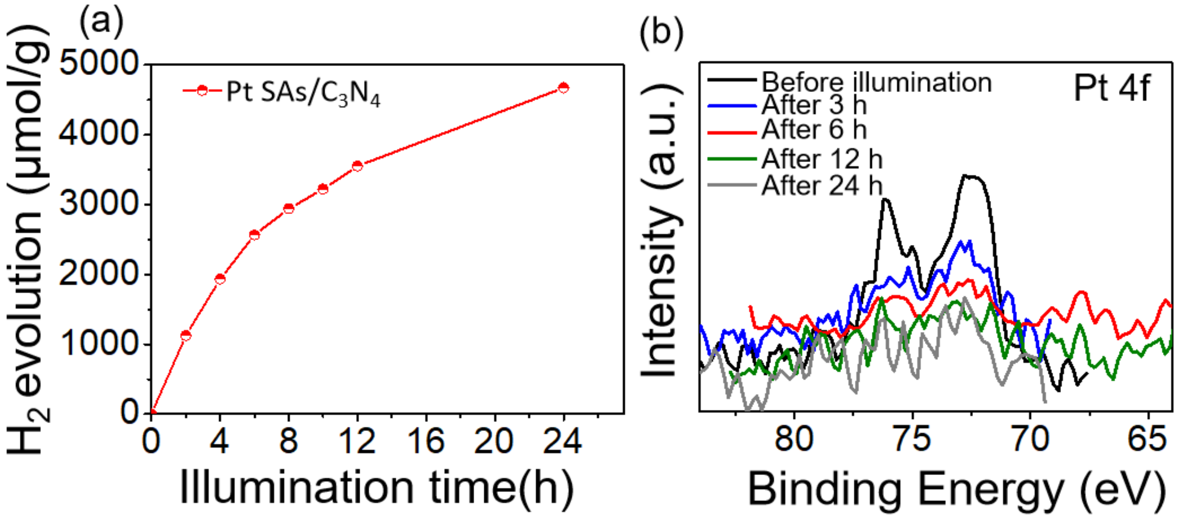

Figure S8. (a) Photocatalytic H_2 evolution of Pt SAs/C_3N_4 after 24 h illumination and (b) Pt 4f XPS spectra of Pt SAs/C_3N_4 before and after illumination.

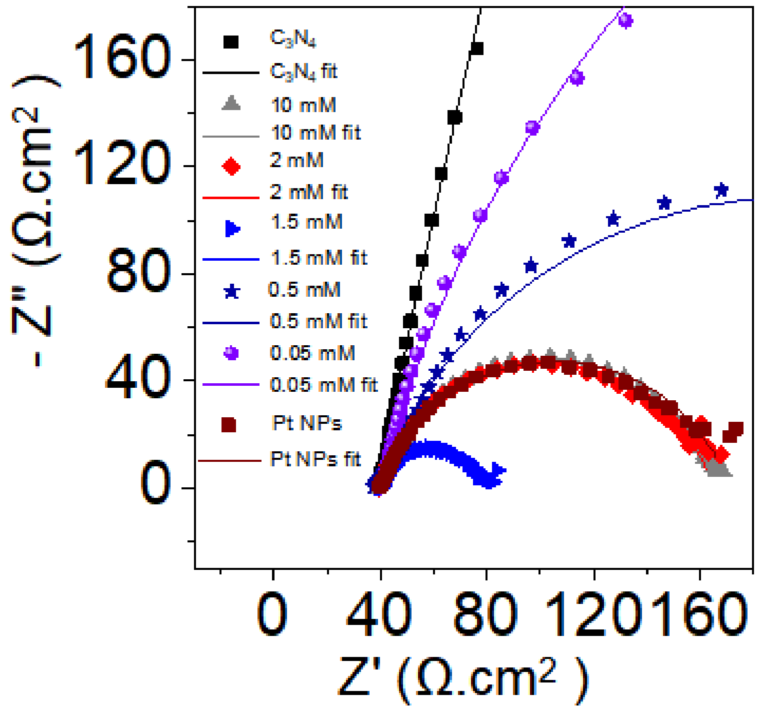

Figure S9. EIS plots of C_3N_4, Pt SAs/C_3N_4 and Pt NPs/C_3N_4 at the voltage -0.5 V (vs.Ag/AgCl) in 0.1 M Na_2SO_4 aqueous electrolyte.

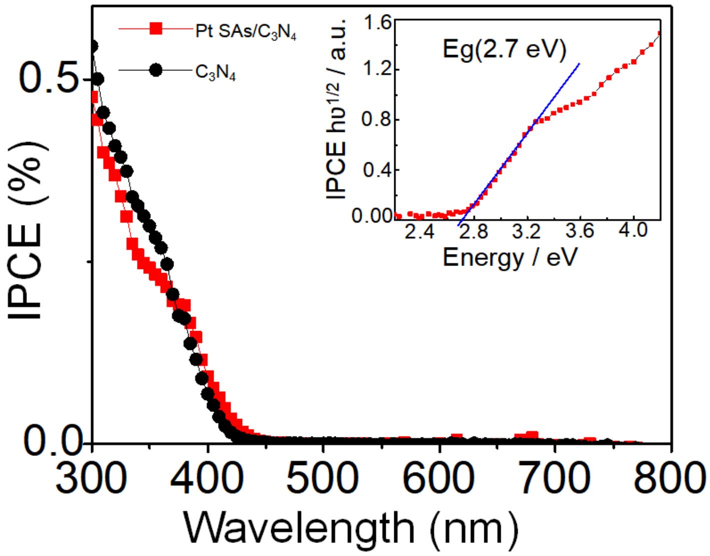

Figure S10. IPCE spectra of bare C_3N_4 and Pt SAs/C_3N_4 measured at a potential of 0.6 V vs Ag/AgCl in a 0.1 M Na_2SO_4 solution in H_2O: Methanol= 90:10 vol. %. The inset shows the evaluation of the band gap from the IPCE spectrum

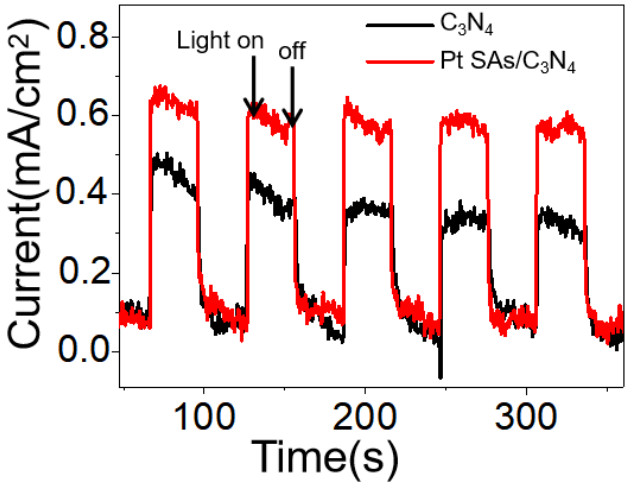

Figure S11. The transient photocurrent of C_3N_4 and Pt SAs/C_3N_4.

Table S1. Pt atomic loading from XPS spectra

**Sample**	10 mM	2 mM	1.5 mM	0.5 mM	0.05 mM	Pt NPs
**Pt loading (at%)**	0.14	0.07	0.05	0.02	-*	0.14

*Below the detection limit.

Table S2. Weight percent (wt%) of Pt from AAS measurement

**Sample**	10 mM	2 mM	1.5 mM	0.5 mM	0.05 mM	Pt NPs
**Pt loading (wt%)**	0.03	0.03	0.02	0.01	0.003	0.15

Table S3. Pt atomic loading from XPS spectra of Pt/ C_3N_4 samples synthesized according to the literature procedures

**Sample**	Ar Annealing[2]	N_2 Annealing[3]	SEA[4]	Sonication[5]	Dark deposition
**Pt loading (at%)**	0.40	0.04	0.06	0.07	0.07

Table S4. Electrical parameters of the C_3N_4 and Pt SAs/C_3N_4 samples extracted from EIS plots by fitting with an equivalent circuit model

**Sample**	**RS (Ω cm^2)**	**Rct (Ω cm^2)**	**Q (s)**	**n**	**Ct (µF cm^{-2})**
**C_3N_4**	38.99	9223.2	0.00076	0.88	991.46
**10mM**	39.55	127.48	0.00053344	0.821414	300.58
**2mM**	39.53	125.18	-	-	356.12
**1.5mM**	39.96	40.488	0.0012389	0.79759	579.72
**0.5mM**	38.179	312.15	0.0018387	0.77458	1564.36
**0.05mM**	40.089	858.12	0.00077125	0.84849	716.45
**Pt NPs**	39.856	130.99	0.0010811	0.79574	654.58

References

(1) Qin, S.; Will, J.; Kim, H.; Denisov, N.; Carl, S.; Spiecker, E.; Schmuki, P. Single Atoms in Photocatalysis: Low Loading Is Good Enough! *ACS Energy Letters* **2023**, *8*, 1209–1214. https://doi.org/10.1021/acsenergylett.2c02801.

(2) Li, X.; Bi, W.; Zhang, L.; Tao, S.; Chu, W.; Zhang, Q.; Luo, Y.; Wu, C.; Xie, Y. Single-Atom Pt as Co-Catalyst for Enhanced Photocatalytic H2 Evolution. *Advanced Materials* **2016**, *28* (12), 2427–2431. https://doi.org/10.1002/adma.201505281.

(3) Zhang, L.; Long, R.; Zhang, Y.; Duan, D.; Xiong, Y.; Zhang, Y.; Bi, Y. Direct Observation of Dynamic Bond Evolution in Single-Atom Pt/C3N4 Catalysts. *Angewandte Chemie International Edition* **2020**, *59* (15), 6224–6229. https://doi.org/10.1002/anie.201915774.

(4) Zuo, Y.; Li, T.; Zhang, N.; Jing, T.; Rao, D.; Schmuki, P.; Kment, Š.; Zbořil, R.; Chai, Y. Spatially Confined Formation of Single Atoms in Highly Porous Carbon Nitride Nanoreactors. *ACS Nano* **2021**, *15* (4), 7790–7798. https://doi.org/10.1021/acsnano.1c01872.

(5) Hu, Y.; Qu, Y.; Zhou, Y.; Wang, Z.; Wang, H.; Yang, B.; Yu, Z.; Wu, Y. Single Pt Atom-Anchored C3N4: A Bridging Pt–N Bond Boosted Electron Transfer for Highly Efficient Photocatalytic H2 Generation. *Chemical Engineering Journal* **2021**, *412*, 128749. https://doi.org/10.1016/j.cej.2021.128749.
